# The Overlooked Microbiome—Considering Archaea and Eukaryotes Using Multiplex Nanopore-16S-/18S-rDNA-Sequencing: A Technical Report Focusing on Nasopharyngeal Microbiomes

**DOI:** 10.3390/ijms24021426

**Published:** 2023-01-11

**Authors:** Carolin Baehren, Anton Pembaur, Patrick P. Weil, Nora Wewers, Frank Schult, Stefan Wirth, Jan Postberg, Malik Aydin

**Affiliations:** 1 Laboratory of Experimental Pediatric Pneumology and Allergology, Center for Biomedical Education and Research, School of Life Sciences (ZBAF), Faculty of Health, Witten/Herdecke University, 58455 Witten, Germany; 2 Clinical Molecular Genetics and Epigenetics, Faculty of Health, Center for Biomedical Education & Research (ZBAF), Helios University Hospital Wuppertal, Witten/Herdecke University, Alfred-Herrhausen-Str. 50, 58448 Witten, Germany; 3 Center for Child and Adolescent Medicine, Center for Clinical and Translational Research (CCTR), Helios University Hospital Wuppertal, Witten/Herdecke University, 42283 Wuppertal, Germany

**Keywords:** archaeome, archaea, eukaryotes, PCR, sequencing, MinION, respiratory diseases

## Abstract

In contrast to bacteria, microbiome analyses often neglect archaea, but also eukaryotes. This is partly because they are difficult to culture due to their demanding growth requirements, or some even have to be classified as uncultured microorganisms. Consequently, little is known about the relevance of archaea in human health and diseases. Contemporary broad availability and spread of next generation sequencing techniques now enable a stronger focus on such microorganisms, whose cultivation is difficult. However, due to the enormous evolutionary distances between bacteria, archaea and eukaryotes, the implementation of sequencing strategies for smaller laboratory scales needs to be refined to achieve as a holistic view on the microbiome as possible. Here, we present a technical approach that enables simultaneous analyses of archaeal, bacterial and eukaryotic microbial communities to study their roles in development and courses of respiratory disorders. We thus applied combinatorial 16S-/18S-rDNA sequencing strategies for sequencing-library preparation. Considering the lower total microbiota density of airway surfaces, when compared with gut microbiota, we optimized the DNA purification workflow from nasopharyngeal swab specimens. As a result, we provide a protocol that allows the efficient combination of bacterial, archaeal, and eukaryotic libraries for nanopore-sequencing using Oxford Nanopore Technologies MinION devices and subsequent phylogenetic analyses. In a pilot study, this workflow allowed the identification of some environmental archaea, which were not correlated with airway microbial communities before. Moreover, we assessed the protocol’s broader applicability using a set of human stool samples. We conclude that the proposed protocol provides a versatile and adaptable tool for combinatorial studies on bacterial, archaeal, and eukaryotic microbiomes on a small laboratory scale.

## 1. Introduction

Until today, only a small fraction of the existing archaea organisms can be cultivated successfully, but this is usually not carried out in clinical routine [1]. The elaboration and optimization of sequencing protocols and primer pairs [2], as well as the increased use of techniques including next-generation sequencing (NGS) [2,3] may provide the characterization of important components within the microbiome, which fails through culturing techniques, particularly for archaea.

Previous studies have already revealed that the influence of archaea in human disease must be elucidated using molecular methods, but it seems to be challenging due to the difficulty in detection, identification, and the selective isolation of these organisms [4].

Thus, primer pair selection is critical. The ideal set of primers targets binding sites that are identical in the 16S-rDNA of all archaea or all bacteria or, respectively, in the 18S-rDNA of all eukaryotes. Since this requirement almost certainly does not exist, primer pairs should cover as many taxa as possible. Pausan and colleagues studied ribosomal sequence variants in different body sites (among others, nose, mouth, skin) by the extensive evaluation of primer pairs, and observed that the combination of definite primers, may be also useful [2]. Moreover, distinct factors may be involved for an impaired cultivation of archaea, e.g., inhibited interspecific material exchange, reduced protein and signaling molecules, as well as growth inhibition by oxidative stress [5,6,7]. The slow growth rate of archaea and their putative low abundance in microbial communities provide a significant problem during cultivation, where a rapid bacterial overgrowth may play a specific role [8,9,10], a failure to monitor their growth due to low frequency, as well as insensitive growth indicators (reviewed in [5]). Not least, possible improper transport conditions and suboptimal in vitro culturing parameters differing from the natural environment of the archaea may lead to incubation failures or the induction of a viable but uncultivable condition (reviewed in [5]).

Usually, the sequence coverage of archaea is low when bacteria-specific primers become applied [11]. Here, Bahram and colleagues designed and validated several improved primer pairs to efficiently amplify the highly phylogenetical diversity of archaeal 16S-rRNA, which outperformed commonly universal primers, as well as current specific primers in terms of specificity and coverage [11]. Primer combinations including SSU1ArF, SSU520R, 340F, SSU666ArR and SSU1000ArR covering the V1 and/or V2 regions resulted in the highest numbers of operational taxonomic units when considering for differences in sequencing depth [11]. For short amplicons, primer pairs such as SSU1ArF + SSU520R and 340F-806rB for V1/V2 and V4/V5 SSU regions of SSU using Illumina^®^ or Ion Torrent NGS platforms were importantly recommended [11].

Due to the variability of the archaeal populations between different samples, the design of primers with high species coverage is challenging, and it seems appropriate that specific primers must be developed and combined to uncover more details on archaeal diversity, particularly in clinical specimens [12]. This is reminiscent of eukaryotic diversity, possibly with emphasis on fungi, where 18S-rRNA primer sets were evaluated in previous works. In conclusion, eight primer pairs presented excellent coverage, including Euk-1422–1440, Euk-1624–1642 and Euk-1629–1647 [13].

Importantly, a significant number of bacterial and fungal taxa remained unrecognized in patients with cystic fibrosis when traditional, cultivation techniques were applied, and a large number of fungal genera was not detected in the human oral cavity by using cultivation techniques (reviewed in [14]).

Hugerth and colleagues defined a set of primer pairs for eukaryotic 18S-rDNA sequencing covering variable regions of the gene, and, e.g., Euk-563–1132, 574–1132, as well as 616–1132 covered the V4 and V5 regions within 18-S [15]. While primer pair 616–1132 succeeded in all tests, however, primer 563 seems to be one of the few primers covering major groups of organisms and can be combined appropriately with primer 1132, which also binds to all major eukaryotic phyla [15]. 

The role of the microbiome in pediatric lung diseases has been extensively studied in the literature. Not only the microbiome, but also the fungal colonization/infection, as well as the archaeome may play a role in disease development or exacerbation. This paper summarizes a precise technical method to better understand the clinical overview of bronchial asthma and wheeze, to develop novel ideas on their pathogenesis and to design personalized diagnostic and therapeutic studies for future approaches. Concerning the aims of this special issue, we believe that fungi and archaea should receive a special focus, because these species may also play an important role in other pediatric lung diseases e.g., cystic fibrosis, primary ciliary dyskinesia, or bronchopulmonary disease. Therefore, the developed method is robust and can be translationally applied to other pediatric lung diseases.

Considering the sparse literature, we therefore tested different primer pairs in terms of their usefulness and, as well as the MinION sequencing technology, which will be addressed in this technical manuscript with the major aim to implement these aspects in disease-related studies.

## 2. Results and Discussion

### 2.1. Proof of Concept and Validation

MinION sequencing resulted several times in the identification of taxa with the airway microbial communities, which were not documented in literature, particularly for archaea. To evaluate the plausibility of the MinION sequencing results, selected samples exhibiting sufficient numbers of mapped reads for specific archaeal taxa-of-interest (TOI) were validated through PCR and subsequent Sanger DNA sequencing. Therefore, we designed TOI-specific primers targeting thymidylate synthase gene (TYMS). As rDNA, the TYMS gene is essential in bacteria, archaea, and eukaryotes, but less conserved between distant taxa. We thus expected that sequence variations in TYMS amplicons occur more frequently and can be used for sequencing validation at a higher taxonomic resolution. Sanger sequencing of TYMS amplicon was successfully applied to confirm the fungus *Malassezia restricta* at the species level, as well as the bacteria *Staphylococcus aureus* and *Corynebacterium propinquum*, and the archaeal *Methanobrevibacter* sp. YE315. However, TYMS turned out not to be suitable for archaea under investigation. Here, we applied again a nested PCR strategy targeting rDNA amplification. Herewith, we could validate the Nanopore-sequencing results for the archaea *Methanobrevibacter* sp. YE315, *Halalkalicoccus jeotgali* B3 and *Natrarchaeobaculum aegyptiacum*. 

A list of the selected primers for these different species can be found in the Supplementary Material (Table S1). PCR conditions are stated below (Table 1).

**Figure 1:**
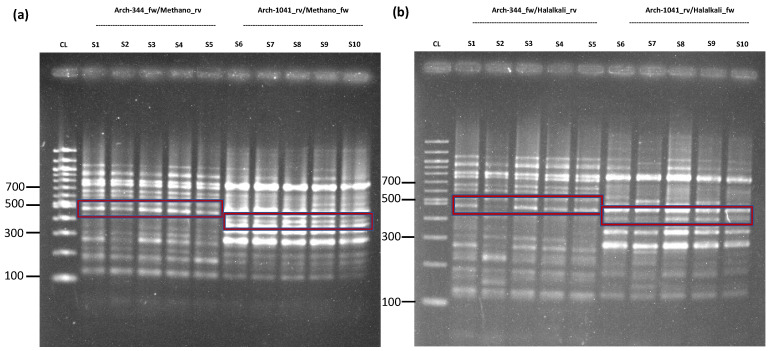
Nested-PCR of *Methanobrevibacter* sp. YE315 and *Halalkalicoccus jeotgali* B3 for further validation. PCR conditions used to be the same as presented in Table 1. (**a**) Primer pair D-Arch-344-fw/*Methanobrevibacter*_rv was used for sample 1 to 5, while D-Arch-1041-rv/*Methanobrevibacter*_fw was used for sample 6 to 10, highlighted in the red box. The amplicon size for sample 1 to 5 is circa 486 bp, and 431 bp for sample 6 to 10. For the first PCR run, the primer pair Arch344-fw/1041-rev with an amplicon size of circa 697 bp for all samples was used. (**b**) The results of the second nested PCR run for *Halalkalicoccus jeotgali* B3. D-Arch-344-fw/*Halalkalicoccus*_rv was used for sample 1 to 5, while D-Arch-1041-rv/*Halalkalicoccus*_fw was used for sample 6 to 10, highlighted in the red box. The amplicon size for sample 1 to 5 was around 487 bp, and circa 432 bp for sample 6 to 10. For the first PCR run, we used primer pair Arch 1st PCR = 344-fw/1041-rev with an amplicon size of approximately 697 bp for all samples, visible results were not detectable here (S = Sample; CL = Control Ladder 100 bp).

Figure 1 presents the results of *Methanobrevibacter* sp. YE315 and *Halalkalicoccus jeotgali* B3. For further Sanger sequencing, sample 1 was chosen due to its matching band and its high count of 29,520 reads for *Methanobrevibacter* sp. YE315 within the MinION sequencing process, and the Sanger sequencing of *Methanobrevibacter* sp. YE315 revealed a successful result. For further validation, we matched some concise sequence sections, including an adequate quality, the results of the database of National Center for Biotechnology Information (NCBI) by Primer-BLAST. There was a clear correlation between the Sanger sequencing results and the information stored in the database regarding *Methanobrevibacter* sp. YE315. Moreover, the selected species-specific DNA sequences from the MinION results successfully matched with the information of the NCBI database. However, the Sanger sequencing for *Halalkalicoccus jeotgali* B3 did not provide successful results, which might possibly be due to the condition of the sample, which had to be diluted for sequencing with distilled water, due to sample shortage, at the time of validation. At the point of MinION sequencing, this sample was still undiluted. Nevertheless, our comparison of the *Halalkaliococcus jeotgali* B3-specific MinION results with the NCBI database showed an average of 88.2% identity for all blasted sequences. Moreover, the sequence tested by Sanger compared to the NCBI database provided a percental identity of 100%.

**Figure 2:**
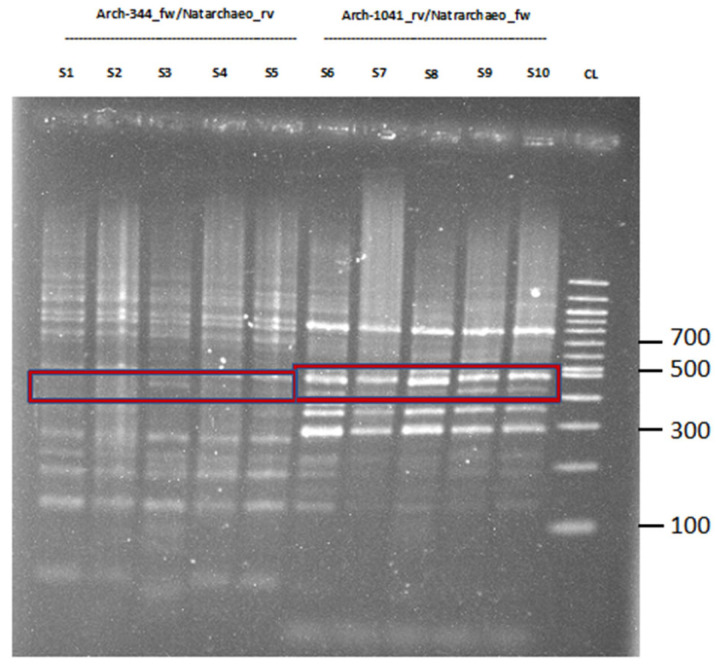
Nested-PCR of *Natrarchaeobaculum aegyptiacum* for qualitative validation. Primer pair D-Arch-344-fw/*Natrarchaeobac*_rv was used for sample 1 to 5, while D-Arch-1041-rv/*Natrarchaeobac*_fw was used for sample 6 to 10, highlighted in the red box (PCR conditions used to be the same as stated in table in Section 3; S = Sample; CL = Control Ladder 100 bp). The amplicon length for D-Arch-344-fw/*Natrarchaeobaculum*_rv was 487 bp, while for D-Arch-1041-rv/*Natrarchaeobaculum*_fw, the length was stated as approximately 432 bp, which was similar to the amplicon length of *Methanobrevibacter* sp. YE315 and *Halalkalicoccus jeotgali* B3. Sample 6 was chosen for further Sanger sequencing. Sanger sequencing of *Natrarchaeobaculum aegyptiacum* was not successful. This sample was already diluted at the time of validation by Sanger sequencing, but not at the time of MinION sequencing, where it provided a total of 7848 reads for *Natrarchaeobaculum aegyptiacum*.

Next, Figure 2 presents the results for *Natrarchaeobaculum aegyptiacum* in the respective position. Our comparison of the *Natrarchaeobaculum aegyptiacum*-specific MinION results with the NCBI database revealed an average of 84.4% identity for all blasted sequences. 

### 2.2. Expanded Testing of the Workflow on Fecal Specimens

**Figure 3:**
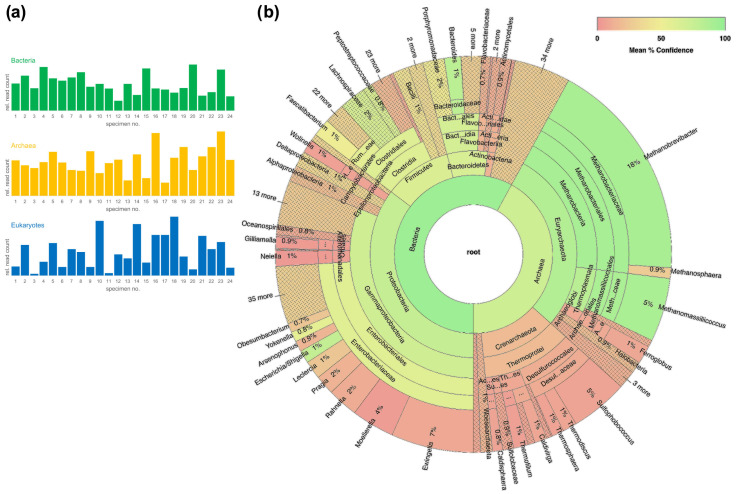
Expanded testing of the workflow on 24 human fecal specimens. (**a**) Relative mapped read counts for bacteria, archaea, and eukaryotes. Mapped reads for all taxa could be identified in all specimens. (**b**) The visualization of the bacterial and archaeal fractions of the cumulative fecal microbial communities was carried out using the 16S Biodiversity Tool of Geneious software (Geneious Prime 2019.2.3). It demonstrates a species-rich diversity of both bacteria and archaea. Importantly, combining archaeal, bacterial, and eukaryotic libraries from a given specimen and using one barcode for all is a strong limitation for mutually quantitative comparisons of mapped reads. Explicitly, quantitative assessments are restricted to the mapped reads for a given taxon, which rely on the identical amplicon used for library preparation. The circular arc defined by the angle alpha correlates with the percentages of taxon-specific reads to total reads, with the outermost concentric circle corresponding to genus level. Due to space limitations, the software tool generates complete labels only if there is enough space for them.

To test the workflows’ broader applicability, we studied the microbial communities using a set of 24 human fecal specimens. Briefly, we found microbial communities composed of bacteria, archaea, and eukaryotes in all specimens. Focusing on bacteria and archaea, a cumulative analysis of all specimens revealed numerous taxa for both taxa (Figure 3b). An important limitation of the workflow of combining archaeal, bacterial, and eukaryotic libraries from a given specimen and using one barcode for all is that mutually quantitative comparisons of mapped reads are illegitimate. Explicitly, quantitative assessments are restricted to the mapped reads for a given taxon, which rely on the identical amplicon used for library preparation. 

### 2.3. Archaeal Cultures for Potential Translational Approaches

Although no archaic pathogens have been clearly identified to date, the possible involvement of these organisms in the development of human diseases is conceivable [12,16,17,18,19]. The presence of a human archaeome has been proven by molecular biology methods, but further translational studies are required in order to investigate whether archaea could influence processes in the human body. These may include important intracellular signaling cascades, communication between cells, as well as the balance in the composition of the microbial communities. Therefore, a culture-based approach could be a promising tool to study direct interactions between archaea and host. A prerequisite to achieve this goal is to isolate and culture archaea candidates in an appropriated context.

Since the development of genetic systems for archaea allowed the application of tools studying, e.g., gene silencing and overexpression, model organisms were established for thermoacidophiles (*Sulfolobus acidocaldarius*), hyperthermophiles (*Pyrococcus furiosus*, *Thermococcus kodakaraensis*), halophiles (*Halobacterium salinarum*, *Haloferax volcanii*), and methanogens (*Methanococcus maripaludis*, *Methanosarcina acetivorans*), which are relatively simple to apply in laboratory (for review, see [20]). 

A general prerequisite for enrichment and isolation is to obtain knowledge about the natural environmental conditions and the stimulation of these conditions in the cultivation method [5].

Methanogenic archaea are predominant in the human gut, and *Methanobrevibacter smithii* is an isolated and cultured candidate [21], which was recently described as a key player in energy harvest, whose absence from the gut microbiome might be connected with severe acute malnutrition [22]. 

Furthermore, *M. smithii* has been detected in the lower respiratory tract [23] and in blood cultures of febrile patients suffering from bacteremia [24]. 

Other methanogenic archaea, which have been successfully cultivated from human samples are *Methanosphaera stadtmaniae* [25], *Methanomassiliicoccus luminyensis* [26] (both originated from human feces), as well as *Methanobrevibacter oralis* isolated from human subgingival plaque [27]. 

Since methanogenic archaea are extremely oxygen-sensitive, special equipment and conditions, e.g., an anaerobic chamber and a hydrogen source are required, and methods must be constantly improved in order to facilitate successful culturing. For example, the recently established co-culturing of *M. smithii* with hydrogen-producing bacteria enables the growth of methane-producing strains without the use of external hydrogen [28]. Other approaches aim to optimize medium by exploring growth enhancing compounds, leading to the development of the highly complex SAB medium [29], which aims for the culture of all methanogenic archaea. 

In a comprehensive investigation based on NGS data from 2017, members of the archaeal DPANN superphylum, especially *Woesearchaeota*, have been identified as highly abundant in broncho-alveolar lavage and lung samples [30]. Due to the lack of cultured isolates, their biology is insufficiently understood, and putative characteristics have been suggested depending on comparative genomic analysis [31].

These microorganisms usually feature small genomes with a reduced metabolic repertoire and therefore depend on the presence of symbionts [32]. For example, *Nanoarchaeum equitans* can only be co-cultured with its obligate host, *Ignicoccus hospitalis*, which provides essential molecules including growth factors, lipids, and amino acids to *N. equitans* [33,34]. Most recently, a thermoacidophilic symbiotic archaeon (ARM-1) from the DPANN superphylum was successfully cultivated using a co-culture system containing *Metallosphaera* sp. AS-7 belonging to the order *Sulfolobales* [35]. 

The cultivation strategy and method, which include the selection of microorganisms based on their size, using selective growth media or selective inhibitors, as well as colony picking from solid media and density-based separation are recently discussed and summarized. Furthermore, other possibilities to obtain enriched cultures are also presented, which consist of motility-based enrichment, dilution-to-extinction, and variation of physicochemical conditions (reviewed in [36]). 

Different approaches aiming to improve the cultivability of archaea have been recently discussed. Since many microorganisms play a role in oxidation/reduction processes and depend on electron transfers for growth, direct interspecies electron transfer (DIET) might be a mechanism that enhances growth of organisms, which are difficult to cultivate [37]. 

Interestingly, Holmes and colleagues reported that the genetically tractable methanogen *Methanosarcina acetivorans* was able to grow via DIET with *Geobacter metallireducens* serving as the electron-donating partner [37].

In order to isolate organisms-of-interest from a mixed sample, cell sorting via droplet-based or microfluidic-based sorters [38,39], as well as fluorescence-activated cell sorting may be helpful techniques [36]. 

## 3. Material and Methods

In order to perform 16S/18S-analyses via MinION Nanopore-sequencing (Oxford Nanopore Technologies (ONT), Oxford, UK), DNA was purified from nasopharyngeal swab specimens prior to PCR amplification and subsequent library preparation via PCR barcoding. We made use of the QIAamp^®^ DNA Mini Kit (Qiagen, Hilden, Germany) and the modified QIAamp^®^ tissue protocol according to manufacturer’s instructions (compare [40]). Purified DNA was quantified using the Promega Quantus fluorometer and the QuantiFluor dsDNA System (Promega, Madison, WI, USA) and, alternatively, using a Nanophotometer P330 (Implen NanoPhotometer^®^, Westlake Village, CA, USA). A detailed protocol is provided in the supplementary material section (Supplementary Materials Tables S1–S3).

Targeting archaeal 16S-rDNA, we sometimes experienced PCR failure using primers Arch-344F and Arch-1041R alone, most probably due to low archaeal DNA concentrations in some specimens. In order to overcome, a nested PCR approach was chosen, where amplification step 1 (Arch-344F and Arch-1041R) was followed by a further amplification step 2 using primers Arch-519F and Arch-786Rtag. 

Table 2 presents the PCR conditions in detail. A list of primer sequences can be found in the Supplementary Material (Table S2).

**Figure 4:**
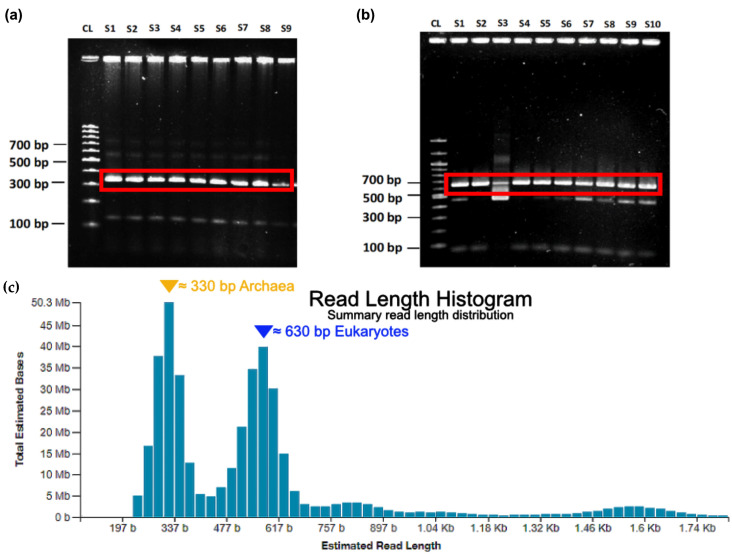
Respective ‘band-of-interest’ for archaea and eukaryotes after PCR. (**a**) A nested PCR run on archaeal DNA resulted in an ‘inner’ amplicon, which was defined by the tagged primer pair Arch-519F/Arch-786Rtag. The amplicon size for tagged archaea was around 330 bp. (**b**) For eukaryotes, a separate PCR run with the primer pair Euk-563F/Euk-1132Rtag was performed. The amplicon-of-interest had an approx. size around 630 bp. (Results highlighted in the red box, S = sample; CL = 100 bp size control ladder). (**c**) Archaeal und eukaryotic tagged amplicon libraries were mixed prior to sequencing. The read lengths distribution chart can be used to supervise nanopore sequencing in real time. The read lengths are mainly determined by the lengths of the tagged amplicon.

Subsequently, the amplificated DNA was semi-qualitatively assessed via agarose gel electrophoresis (Figure 4). The expected amplicon size for eukaryotes was approximately 630 bp and, respectively, approximately 330 bp for the archaea after PCR 2. Nested PCR targeting archaeal 16S-rDNA resulted in improved amplification success. For eukaryotes, a single PCR step, which included 30 cycles provided satisfactory results (Figure 4b). In practice, some weaker additional bands appeared frequently. However, these additional bands turned out not to interfere with the following workflow.

Next, the PCR barcoding kit (PBAC96_9069_v109_revO_14Aug2019, ONT) was used for sequencing library preparation. The amplicon-specific PCR was performed using tagged primers, which contain a barcode and 5′ tags, which facilitate the ligase-free attachment of Rapid Sequencing Adapters. The kit contains 12 different barcodes for multiplex sample preparation. The tag provides a binding side for the primers of the following multiplexing PCR. Before multiplexing, a clean-up step with Agencourt AMPureXP magnetic beads (Beckmann Coulter, Brea, CA, USA) was performed. Through PCR, we multiplexed 12 specimens using the PCR barcoding kit primers. Thereby, archaeal, and eukaryotic samples from each individual nasopharyngeal swab specimen received the same barcode for simultaneous sequencing and bioinformatic demultiplexing. After multiplexing, a second clean-up was performed. In detail, as also stated in the protocol, we added 36 µL beads and applied an external magnetic field for 5 min. The fluidic supernatant was discarded. Next, two washing steps with 70% ethanol were performed by adding 150 µL 70% ethanol and afterwards the fluidic supernatant was discarded, and the dried pellet was resuspended in 15 µL nuclease-free water. The concentration was measured by fluometry, or photometry as described above. Next, the libraries were pooled considering equal molarity for each multiplexed sample. The DNA repair and end-preparation step were then carried out, sequencing adapters were ligated, and another clean-up step using Agencourt AMPureXP magnetic beads was carried out. For library loading onto the flow cell, we deviated from the manufacturer’s protocol, since we determined that 500 fmol of the final library were sufficient to achieve the required sequencing depth.

The sequencing was performed on a MinION MK1B device, and the base-calling was done afterwards using Guppy Version 3.5.1. The downstream analysis was carried out using the Epi2Me platform, provided by ONT, where the WIMP (‘What‘s in my pot’) Workflow (v2021.09.09) was chosen. Alternatively, the 16S Biodiversity Tool (Geneious, Auckland, New Zealand) provided a useful visualization tool. 

**Figure 5:**
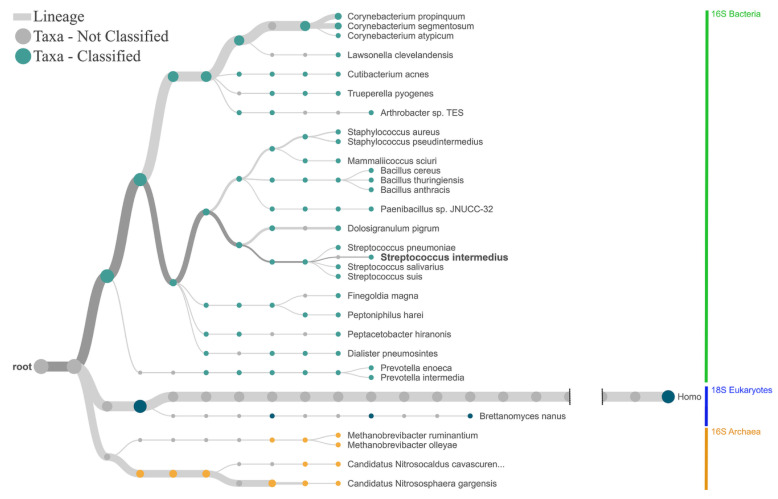
Exemplary output of the WIMP workflow. The workflow outputs a phylogenetic tree, where the thickness of the branches correlates with the number of mapped reads. The output can be filtered by barcode or organism. The taxonomic resolution of the output of the WIMP workflow can be adjusted by setting a cutoff to the minimal number of reads per represented organism. The results can be also restricted to specific barcodes.

The results of the WIMP Workflow were analyzed to determine the differences in length distribution of the reads between superkingdoms. Therefore, a python script was used to determine the length of each read from the FastQ files. The SQL-Database approach with the results of the WIMP workflow was utilized to separate the superkingdoms. From the resulting dataset, a virtual gel was created and compared to the existing agarose gels. The comparison illustrated that most of the bacterial amplicons were produced by the primers that were used to amplify the eukaryotic sequences (Figure 5). Notably, as illustrated in Figure 5, human DNA is an unavoidable source of contamination that affects library manufacturing, ultimately at the expense of sequencing read depth. To minimize this effect, we have attempted to minimize this side effect by depleting human DNA. The principle of the NEBNext Microbiome DNA Enrichment kit (New England Biolabs, Ipswich, MA, USA) is the specific digestion of CpG-methylated DNA, which is not found in prokaryotes. However, comparison of the workflow with or without this step did not lead to any significant changes in the sequencing results, i.e., no detectable depletion of human DNA contaminants.

We provide an online protocol with detailed instructions, which is available as follows: https://www.protocols.io/view/characterization-of-the-archaeome-bacteriome-and-e-ce7cthiw (accessed on 16 September 2022).

## 4. Conclusions

While most of the current research regarding microbial communities in and on the human body focuses on bacteria, studies on archaea and eukaryotes are still under-represented. In particular, the composition and function of the human archaeome is insufficiently understood. 

The aim of this work was to demonstrate the importance of the right choice of primers for the detection of archaea and eukaryotic genes in total DNA samples to overcome difficulties in the amplification of such sequences. Furthermore, using the MinION sequencing device from Oxford Nanopore Technologies proposes a promising approach to shed new light on the composition of the archaeome and mycobiome of the human nasopharynx in pediatric respiratory disease, such as asthma. Finally, we present an outlook on possible future perspectives for translational studies on the influence of archaea on the human body using culture-based methods, in particular in the context of pediatric lung diseases.

Therefore, total DNA was isolated from nasopharyngeal swab specimens. Subsequently, specific sequences of genes encoding the archaea 16S-rRNA and the eukaryotic 18S-rRNA were amplified by PCR. In order to increase sensitivity and specificity, a nested approach using the superior performing primer pair combination Arch-344F-1041R/Arch-519F-786Rtag [2] was used for the amplification of the sequence covering the archaea 16-S-rRNA gene. For the identification of eukaryotic sequences, a defined region of the 18-S-rRNA gene was amplified using the primer pair Euk-563F-Tag/Euk-1132R-Tag [15]. The amplicons were purified using magnetic beads and pooled DNA libraries were sequenced using a MinION MK1B device. During evaluation of this PCR protocol, the outcome of several studies on the design of specific primers targeting archaea and eukaryotes could be confirmed. Both PCR experiments revealed additional bands that are most likely bacterial fragments, confirming the difficulty to obtain amplicons solely originating from the archaeal and eukaryotic domains. Alternatively, the position of the binding sites of the primers differs based on the organism could be another explanation for the presence of additional bands, which differ in size and intensity.

The innovative Nanopore sequencing technique could be a powerful and time saving tool to distinguish the desired domain-specific products from bacterial impurities and to exactly link the amplicons to a specific source of organism. 

Notably, the improvement of culturing techniques and subsequent translational research based on culturing are essential to uncover the effect of the respective organism on human cells and tissues, and therefore investigate the influence of the human archaeome and mycobiome on health. 

## Figures and Tables

**Table 1:** PCR conditions for archaea used for validation. Primer S-D-Arch-0344-fw and S-D-Arch-1041-rev were used for the 1st PCR. The PCR volume was 20 µL. For PCR 1°, 4 µL RNase-free water, 10 µL GoTaq G2 Hot Start Green Master Mix (Promega, Madison, WI, USA), 4 µL primer mix, and 2 µL DNA were used. In PCR 2°, 2 µL of the before mentioned PCR were added to 10 µL RNase-free water, 10 µL GoTaq G2 Hot Start Green Master Mix, and 4 µL primer mix.

Targets	Archaea	
(Nested) PCR	PCR 1	PCR 2
Primer pair	Arch-344F/Arch-1041R	TOI-specific (Table S1)
1st Denaturation	95 °C 3 min	95 °C 3 min
Number of cycles	30	28
Denaturation	95 °C 30 s	95 °C 3 s
Annealing	55 °C 30 s	55 °C 30 s
Elongation	72 °C 30 s	72 °C 30 s
Final Extension	72 °C 5 min	72 °C 5 min

**Table 2:** PCR conditions for archaea and eukaryotes used in this approach. For denaturation, annealing, and elongation, the corresponding time and temperature are stated below. A nested PCR was performed for the archaea. PCR reaction volume was 25 µL. In detail for PCR 1°, 8 µL RNase-free water, 12.5 µL Q5 High Fidelity Polymerase 2× Master Mix (New England Biolabs, Ipswich, MA, USA) and 2 µL mix of Arch-344F and Arch-1041R were added (final concentration for all primers was 10 µM). PCR 2° was prepared by adding 9.5 µL RNase-free water, 12.5 µL Q5 Polymerase Master Mix and 2 µL mix of Arch-519F and Arch-786Rtag. For eukaryotes, conditions were reminiscent of archaeal PCR 1, with archaeal-specific primers being replaced by primer mix Euk-563F and Euk-1132Rtag.

Targets	Archaea		Eukaryotes
(Nested) PCR	1	2	1
Primer pair	Arch-344F/Arch-1041R	Arch-519F/Arch-786Rtag	Euk-563F/Euk-1132Rtag
Amplicon size	697 bp	330 bp	630 bp
1st Denaturation	95 °C 3 min	95 °C 3 min	95 °C 3 min
Number of cycles	30	28	30
Denaturation	95 °C 30 s	95 °C 3 s	95 °C 30 s
Annealing	55 °C 30 s	55 °C 30 s	55 °C 30 s
Elongation	72 °C 30 s	72 °C 30 s	72 °C 30 s
Final Extension	72 °C 5 min	72 °C 5 min.	72 °C 5 min.

## Data Availability

The data presented in this study are available on reasonable request from the corresponding or first authors. An online protocol with detailed instructions is available: https://www.protocols.io/view/characterization-of-the-archaeome-bacteriome-and-e-ce7cthiw (last 22 September 2022).

## Supplementary Materials

The following supporting information can be downloaded at: https://www.mdpi.com/article/10.3390/ijms24021426/s1. Reference [40] is cited in supplementary material.
