# Supplementary material for: The Overlooked Microbiome—Considering Archaea and Eukaryotes Using Multiplex Nanopore-16S-/18S-rDNA-Sequencing: A Technical Report Focusing on Nasopharyngeal Microbiomes
Source: Int J Mol Sci. 2023 Jan 11;24(2):1426. doi: 10.3390/ijms24021426 (PMC9863950; doi:10.3390/ijms24021426)
Supplement: Supplementary file 1 [file ijms-24-01426-s001.zip › ijms-1959560-supplementary.pdf]

Supplementary Material

Supplementary Table S1. Primer selection for validation of selected taxa using TYMS or rDNA amplicons.

No.	Primer Name	Sequence
1	TYMS-*Malassezia*_fw	5′-cccgtgccatatcctctgtc -3′
2	TYMS-*Malassezia*_rv	5′-agttccacatcttcagggcg -3′
3	TYMS-*Staphylococcus*_fw	5′-tcttgctgggaacgatgtcc -3′
4	TYMS-*Staphylococcus*_rv	5′-tgggcatcaattgcgtttcg -3′
5	TYMS-*Corynebact*_fw	5′- tatcgcttcctacgcactgct-3′
6	TYMS-*Corynebact*_rv	5′-cgtcgacggagatatcggaaa -3′
7	S-D-Arch-0344-fw	5′-acggggygcagcaggcgcga-3′
8	S-D-Arch-1041-rev	5′-ggccatgcaccwcctctc-3′
9	*Methanobrevi*_nest_fw	5′-gaattgctggagatactatta-3′
10	*Methanobrevi*_nest_rv	5′-cttcccttcggcactgagac-3′
11	*Halalkali*_nest_fw	5′-acgtccggcggaaaccagt-3′
12	*Halalkali*_nest_rv	5′-cttccctacggcacagcact-3′
13	*Natrarch*_nest_fw	5′-gcgtccggtggaaactgcg-3′
14	*Natrarch*_nest_rv	5′-cttccctacggcacatcaca-3′

Supplementary Table S2. Primer selection for archaea and eukaryotes. Stated names refer to the names used in the original papers, stated primer names refer to the original manufacturer identification.

No.	Name	Primer name	Sequence
1	344F	S-D-Arch-0344-fw	5′-acggggygcagcaggcgcga-3′
2	1041R	S-D-Arch-1041-rev	5′-ggccatgcaccwcctctc-3′
3	519F	Arch-519F-Tag	5′-tttctgttggtgctgatattgccagcmgccgcggtaa-3′
4	786R	Arch-786R-Tag	5′-acttgcctgtcgctctatcctcggactacvsgggtatctaat-3′
5	563F	Euk-563F-Tag	5′-tttctgttggtgctgatattgcgccagcavcygcggtaay-3′
6	1132R	Euk-1132R-Tag	5′-acttgcctgtcgctctatcttcccgtcaatthcttyaart-3′

Supplementary Table S3. Modified DNA purification protocol for nasopharyngeal swab specimen according to biological fluids protocol and DNA Purification from Tissues protocol in QIAamp® DNA Mini and Blood Mini Handbook 05/2016 by QIAGEN (also adapted from [40]).

a) Centrifugation of bacteria pellet: 10 min at 5000× *g* (7500 rpm)
b) Resuspension of the pellet in 180 μL Buffer ATL
c) Adding 20 μL proteinase K, vortexing, incubation at 56 °C until lysis is completed. Occassionally vortexing.
d) Briefly centrifugation of the sample.
e) Adding 200 μL Buffer AL, pulse-vortex afterwards (15 s), incubation at 70 °C (10 min). Short centrifugation of the sample.
f) Adding 200 μL ethanol (96–100%), mixing by pulse-vortexing (15 s). Afterwards, short centrifugation of the sample.
g) Transfering mixture (including precipitate) to the QIAamp Mini spin columns. CAVE: without wetting the rim. Centrifugation for 1 min at 6000× *g* (8000 rpm). Replace the QIAamp Mini spin columns into a clean 2 mL collection tube, discard the tube with the filtrate.
h) Adding 500 μL Buffer AW1, CAVE: without wetting the rim. Centrifuge for 1 min at 6000× *g* (8000 rpm). Replace the QIAamp Mini spin columns into a clean 2 mL collection tube, discard the tube with the filtrate.
i) Adding 500 μL Buffer AW2 to the QIAamp Mini spin column without wetting the rim. Close the column and centrifuge for 3 min at full speed (20,000× *g*)
j) Replace QIAamp Mini spin columns into a new 2 mL collection tube. Discard the tube with the filtrate and centrifuge for 1 min at full speed (20,000× *g*)
k) Place QIAamp Mini spin columns to a new 1.5 mL microcentrifuge tube. Discards the tube with the filtrate and add 100 μL Buffer AE/distilled water. Incubate at room temperature for 5 min and centrifuge for 1 min at 6000× *g* (8000 rpm).
l) Repeat step k): Repeat the flowthrough of the previous step to the same Mini Spin column, incubate at room temperature for 5 min, and centrifuge for 1 min, at 6000× *g* (8000 rpm)
m) Instead of 200 μL, as provided in QIAamp® Handbook, we used 100 μL in step k and incubated for 5 min, instead of 1 min, because extended incubation generally increased DNA yield, in our opinion. Afterwards, in step l, we used the same 100 μL and transferred back to the columns and incubated for further 5 min.
